# Clomiphene citrate-induced visual hallucinations: a case report

**DOI:** 10.1186/s13256-017-1228-0

**Published:** 2017-03-06

**Authors:** Ramesh Venkatesh, Gaganjeet Singh Gujral, Prachi Gurav, Shailja Tibrewal, Umang Mathur

**Affiliations:** Department of Retina and Vitreous, Shroff’s Charity Eye Hospital, 5027, Kedarnath Road, Daryaganj, New Delhi, 110002 India

**Keywords:** Clomiphene citrate, Polycystic ovarian syndrome, Side effects, Palinopsia

## Abstract

**Background:**

Polycystic ovary syndrome is a common cause of chronic anovulation and infertility in otherwise healthy fertile couples. Clomiphene citrate is used as a first-line ovulation induction therapy in patients with polycystic ovary syndrome. Clomiphene citrate can cause both systemic and ocular side effects. We report a rare side effect of illusory palinopsias in a patient with polycystic ovary syndrome treated with ovulation induction therapy with clomiphene citrate, and emphasize the need for gynecologists and their patients to be aware of this rare ocular side effect.

**Case presentation:**

A 30-year-old Asian woman complained of persistent visual afterimages following treatment with 100 mg clomiphene citrate for anovulation. Her symptoms started on the fourth day after commencing the treatment and would last for 5 to 10 minutes. Similar visual symptoms were noted during her second cycle of treatment with clomiphene citrate. The severity of her symptoms reduced following the stoppage of the medication; however, the symptoms have persisted for more than 1 year since she stopped taking the drug.

**Conclusions:**

Clomiphene citrate can cause disturbing illusory palinopsias. These afterimages persist even after stopping the infertility medication. It is a side effect not frequently seen by gynecologists or ophthalmologists. Gynecologists should make their patients aware of this rare ocular side effect when their patients start treatment with clomiphene citrate for infertility.

## Background

Clomiphene citrate (CC) is a common selective estrogen receptor modulator (SERM) used in ovulation induction therapy in patients with polycystic ovary syndrome (PCOS) that usually results in chronic anovulation [1]. We report a case of illusory palinopsias with CC in a patient with PCOS and emphasize the need for the gynecologists to make their patients aware of this rare ocular side effect of the drug.

## Case presentation

A 30-year-old Asian woman diagnosed as having PCOS underwent ovulation induction therapy with CC 100 mg tablet daily for 5 consecutive days from day 5 to 9 of her menstrual cycle. On the fourth day of commencing the treatment, she developed visual disturbances and characteristically described them as noting same color afterimages of non-illuminated objects, shadow of a person moving in front of her, some kind of flash on moving from a poorly illuminated zone to a strongly illuminated zone, and some waves in vision in broad daylight. The symptoms would occur five to seven times in a month. The duration of these visual symptoms would last for approximately 5 to 10 minutes. Her symptoms would gradually reduce after she completed her treatment course for 5 days. Her symptoms were not accompanied by other ocular symptoms such as pain, redness, photophobia, or decrease in vision. She gave no past or family history of migraine. A second course with ovulation induction therapy again with 100 mg CC was prescribed to her in the following month. She developed similar visual symptoms 4 days after starting the treatment. She conceived after taking two cycles of CC. One year post-delivery, she visited an ophthalmologist at a tertiary eye hospital with complaints of persistence of symptoms; although the severity and frequency of symptoms were much less than when she was under treatment with CC. On ophthalmic examination, her best-corrected visual acuity in both eyes was 6/6, N6. Anterior and posterior segment examinations of both eyes were normal. Brain magnetic resonance imaging (MRI) was normal. A probable diagnosis of CC-induced illusory palinopsia was made. She was counseled regarding her condition and was asked to follow-up at a regular interval of every 6 months.

## Discussion

Palinopsia is a visual disturbance characterized by a persistent recurrence of a visual image after the stimulus has been withdrawn. Palinopsia is broadly grouped into two categories: illusory palinopsias and hallucinatory palinopsias [2]. Hallucinatory palinopsias are due to posterior cortical lesions. The afterimages described are formed, long-lasting, and high resolution. Illusory palinopsias are caused by migraines, head trauma, prescription drugs, or hallucinogen persisting perception disorder. In illusory palinopsias, the afterimages described are affected by ambient light and motion and are unformed, indistinct, or low resolution. In our case, the patient noted positive afterimages which were indistinct, poorly formed, and low resolution. These symptoms were very similar to that described in illusory palinopsias. An MRI of her brain did not show any neurological lesions. Her visual symptoms were not followed by migraine-like headache. Hence, the symptoms described are classical of illusory palinopsias. Illusory palinopsias are caused by diffuse neuronal pathology such as global alterations in neurotransmitter receptors. CC, a SERM, is used as the first line of therapy for pharmacological ovulation induction. CC is characterized by agonistic properties when endogenous estrogen levels are low, and acts as a competitive antagonist when levels are high. Depletion of estrogen receptors in the hypothalamus results in normalization of gonadotropin-releasing hormone secretion, leading to optimization of secretion of pituitary follicle-stimulating hormone and hence follicular development and ovulation [3]. Yilmaz *et al*. [4] have shown different patterns of visually evoked potential latencies during different phases of the menstrual cycle. The latencies are reduced during the follicular and ovulatory phase of the menstrual cycle whereas they are increased during the ovulatory phase of the cycle [4]. Estrogen inhibits the synthesis of gamma-aminobutyric acid, an important inhibitor neurotransmitter in the cerebral and visual cortexes, and is involved in the genesis of visually evoked potentials. The inhibition of gamma-aminobutyric acid reportedly increases the excitatory effect on the striate cortex [5]. Thus, estrogen can directly or indirectly stimulate the visual cortex, thus triggering the development of visual hallucinations. CC causes ocular side effects such as central retinal vein occlusion, irreversible palinopsias, optic neuropathy, anterior uveitis, and maculopathy [6–10]. Purvin [7] described visual hallucinations in three women treated for infertility with CC for 4 to 15 months. Despite stopping treatment, these women remained symptomatic for a prolonged period of time. In our case, the symptoms occurred with the intake of the drug in the first month. The symptoms started as early as 4 days following treatment and have persisted for more than 1 year following the cessation of the drug. However, the frequency and severity of symptoms reduced after stopping the drug. We believe that the visual symptoms described by our patient were secondary to the use of CC.

## Conclusions

To conclude, CC can cause disturbing visual palinopsias and afterimages. Although previously reported, this case describes a rare ocular side effect caused by CC, not frequently seen either by ophthalmologists or gynecologists. Gynecologists and/or infertility experts should educate their patients regarding these possible ocular symptoms.

## Abbreviations

CC: Clomiphene citrate
MRI: Magnetic resonance imaging
PCOS: Polycystic ovary syndrome
SERM: Selective estrogen receptor modulator
